# Increased B3GALNT2 in hepatocellular carcinoma promotes macrophage recruitment via reducing acetoacetate secretion and elevating MIF activity

**DOI:** 10.1186/s13045-018-0595-3

**Published:** 2018-04-04

**Authors:** Tianxiao Yang, Yilin Wang, Wenjuan Dai, Xixi Zheng, Jing Wang, Shushu Song, Lan Fang, Jiangfan Zhou, Weicheng Wu, Jianxin Gu

**Affiliations:** 1 Department of Biochemistry and Molecular Biology, School of Basic Medical Sciences, Fudan University, Shanghai, China; 2 Key Laboratory of Glycoconjugate Research Ministry of Health, School of Basic Medical Sciences, Fudan University, Shanghai, China; 3 Department of Hepatic Surgery, Fudan University Shanghai Cancer Center, Fudan University, Shanghai, China; 4 Department of Oncology, Shanghai Medical College, Fudan University, Shanghai, China; 5 Shanghai Tenth People’s Hospital of Tongji University, School of Medicine and School of Life Science and Technology, Tongji University, Shanghai, China; 6 Department of Hepatobiliary, The First Affiliated Hospital of Wenzhou Medical University, Wenzhou, Zhejiang China

**Keywords:** Hepatocellular carcinoma, B3GALNT2, Macrophage recruitment, Acetoacetate, MIF

## Abstract

**Background:**

Hepatocellular carcinoma (HCC) ranks as the sixth most prevalent cancer and the third leading cause of tumor-related death, so it is urgently needed to discover efficient markers and targets for therapy. β-1,3-*N*-acetylgalactosaminyltransferase II (B3GALNT2) belongs to the β-1,3-glycosyltransferases (b3GT) family and has been reported to regulate development of both normal and tumor tissues. However, studies on the functions of B3GALNT2 in cancer are quite limited. Here we investigated the potential role of B3GALNT2 in HCC progression.

**Methods:**

Western blot, qPCR, and immunohistochemistry assays were performed to quantify the relative expression of B3GALNT2 in HCC. The functions of B3GALNT2 in tumor progression were evaluated in HCC cell lines and nude mice. Metabolomics analysis was applied to detect alternatively expressed small molecules. Enzyme activity assays were employed to determine the tautomerase activity of macrophage inhibitory factor (MIF).

**Results:**

For expression analysis, higher levels of B3GALNT2 were observed in tumor tissues compared with adjacent normal tissues, and upregulation of B3GALNT2 correlated with increased tumor size and worse overall survival. Changing levels of B3GALNT2 did not influence cell viability in vitro but promoted tumor growth via enhancing macrophage recruitment in vivo. Furthermore, acetoacetate was identified as a key molecule in B3GALNT2-mediated macrophage recruitment. Mechanistically, B3GALNT2 downregulated expression of enzymes involved in acetoacetate-related metabolism, and reduction of acetoacetate revived MIF activity, thus promoting macrophage recruitment.

**Conclusions:**

This study evaluated B3GALNT2 as a tumor marker in HCC and revealed functions of B3GALNT2 in metabolic transformation and microenvironmental remodeling in HCC. Mechanistically, B3GALNT2 reduced expression of some metabolic enzymes and thus downregulated levels of secreted acetoacetate. This relieved the activity of MIF and enhanced macrophage recruitment to promote tumor growth.

**Electronic supplementary material:**

The online version of this article (10.1186/s13045-018-0595-3) contains supplementary material, which is available to authorized users.

## Background

Hepatocellular carcinoma (HCC) ranks as the sixth most prevalent cancer and the third leading cause of tumor-related death [1, 2]. Despite huge developments in HCC diagnosis and treatment, 5-year survival rates of most HCC patients remain dismal [3]. Therefore, it is urgently needed to discover efficient markers and therapeutic targets for HCC. Emerging evidence has proven that abnormal glycosylation in HCC is closely associated with cancer progression. Some glycosylation modifications have been reported to promote HCC tumor growth, angiogenesis, and metastasis, and some of them are capable of predicting the prognosis of HCC patients [4]. However, due to instability of this glycosylation and the specialized equipment required for detection, it is still hard to use these as rapid markers for clinical application. Abnormal glycosylation usually results from aberrant expression of glycosyltransferases that are relatively stable in tissues and easy to detect [4, 5]. Therefore, some of them like *N*-acetylglucosaminyltransferase V (GnT-V), *N*-acetylglucosaminyltransferase III (GnT-III), and α1-6 fucosyltransferase (a1-6FT) have been used as tumor markers and therapeutic targets in HCC [6–9].

Macrophages originate from immature monocyte in the bone marrow and migrate throughout the entire body through the circulation. Final differentiation occurs in tissues to form macrophages, including Kupffer cells which are found in the liver. It is already known that all kinds of macrophages coexist in tumors, but recruited macrophages may account for the majority of tumor-associated macrophages (TAMs). Peripheral blood monocytes from the bone marrow are recruited and differentiate into TAMs in response to chemokines and growth factors in the tumor microenvironment [10, 11]. TAMs promote solid tumor development through providing factors which can establish a pre-malignant niche and enhance metastasis. TAMs may also play a role in forming pre-metastatic niches in organs where the tumor will eventually metastasize [12]. There is also evidence that TAMs are closely associated with formation of stem-like cells in human cancers [13]. Dysregulation of glycosyltransferases has been reported to regulate the functions of TAMs [14], but whether some glycosyltransferases influence TAM recruitment remains to be elucidated.

β-1,3-*N*-acetylgalactosaminyltransferase II (B3GALNT2) belongs to the β-1,3-glycosyltransferases (b3GT) family, consisting of β-1,3-galactosyltransferases (B3GALT), β-1,3-*N*-acetylglucosaminyltransferases (B3GNT), and β-1,3-*N*-acetylgalactosaminyltransferases (B3GALNT). B3GALNT2 efficiently adds *N*-acetylgalactosamine (GalNAc) on both N-glycans and O-glycans by β-1,3-linkage and generates GalNAcb1 → 3GlcNAcb1-R structure [15]. B3GALNT2 has been reported to regulate the development of both normal tissues and tumor tissues [16]. Upregulation of B3GALNT2 in breast cancer predicts poor prognosis [17]. Since knockdown of B3GALNT2 in zebrafish leads to degeneration of the extracellular matrix [16], B3GALNT2 might also exert functions in cancer progression via altering secretion or remodeling the extracellular environment. However, studies on the functions of B3GALNT2 are quite limited. Whether and how B3GALNT2 functions in HCC remain to be elucidated.

In this study, we investigated expression of B3GALNT2 in HCC and analyzed its potential role in HCC progression. Our study also determined how B3GALNT2 remodels the tumor microenvironments to promote tumor growth.

## Methods

### Hepatocellular carcinoma patient samples

Usage of human pathological tissues and clinical data was approved by the Ethics Committee at the Shanghai Cancer Center of Fudan University (Shanghai, China; approval no. 050432-4-1212B). Written consent for all patients conformed to the ethical guidelines of the Helsinki Declaration. A total of 139 patients with primary HCC resected between 2010 and 2012 in the Department of Hepatic Surgery, Shanghai Cancer Center of Fudan University (Shanghai, China) were collected. None of the patients had received pre-operative therapy. Clinical tumor stages were determined according to the TNM classification system of International Union against Cancer. Follow-up was done until December 9, 2016. These patients were followed every 3 months. The median follow-up was 33.3 months (ranging from 0.8 to 60.4 months). Among all of the primary tumor specimens, 24 were used for Western blot and quantitative real-time PCR assays.

### Cell culture

All HCC cell lines, human THP1 cells, and mouse RAW264.7 cells were obtained from the Cell Bank of Type Culture Collection of the Chinese Academy of Sciences (Shanghai, China) and cultured in Dulbecco’s Minimum Essential Medium (DMEM) supplemented with 10% fetal bovine serum at 37 °C in a humidified atmosphere containing 5% CO_2_. Fetal bovine serum and DMEM culture media were purchased from Sigma (St. Louis, MO, USA). The THP1 cell line was maintained in RPMI 1640 medium supplemented with 10% FBS and 2 mmol/L L-glutamine. THP1 cells were differentiated using 200 nM phorbol-12-myristate-13-acetate (PMA, Sigma-Aldrich) for 3 days.

### Plasmid construction

The cDNA encoding B3GALNT2 was obtained by PCR and was inserted into the pCMV-Flag vector (Sigma, St. Louis, MO, USA). The sequence of the shRNA inserted in the pENTR vector (Thermo, USA) was as follows: B3GALNT2: 5′-CACCGGTCATATAATTGTGTGTTTACGAATAAACACACAATTATATGACC-3′, BDH2: 5′-CACCGGAACAGTTGATACGCCATCTCGAAAGATGGCGTATCAACTGTTCC-3′, and MIF: 5′-TCGAGGACACCAACGTGCCCCGCGCTTCAAGAGAGCGCGGGGCACGTTGGTGTCTTTTTTA-3′. Transfections were performed with Lipofectamine 3000 (Life Technologies, CA, USA), according to the manufacturer’s instructions. Stable cell lines were generated with G418 (200 μg/mL) in the medium.

### Cell viability assay

Cell viability was quantified with a Cell Counting Kit-8 (CCK-8) (Dojindo, Japan), according to the manufacturer’s instructions. The cells were plated at a density of 3000 cells per well in 96-well plates. The CCK-8 assays were assessed by measuring the absorbance at 450 nm.

### Cell cycle and apoptosis assay

Cycle arrest and apoptotic cells were detected by flow cytometric analysis. Cells were collected by trypsinization and washed twice with PBS. For cell cycle assay, the collected cells were stained with propidium iodide (PI) using a Cell Cycle Staining Kit (Lianke Bio, Hangzhou, China). Cellular apoptosis was determined using PE Annexin-V Apoptosis Detection Kit I (BD Biosciences, CA, USA). Stained cells were assessed by flow cytometry and the data were analyzed by FlowJo software (TreeStar, Ashland, OR, USA).

### Transwell assay

Transwell invasion was assessed using 8-μm transwell filters (Milliporem, Billerica, MA, USA) in a 12-well plate. The bottom of the transwell chamber was coated with BD Matrigel Basement Membrane Matrix (BD Biosciences, San Diego, CA, USA). Macrophages were added into the upper chamber containing basic culture medium without serum, and the lower chamber was filled with HCC tumor cell lines in serum-free culture medium. Macrophage infiltration was determined 48 h later. Cells on the upper side of the chamber were removed from the surface of the membrane by scrubbing, and cells on the lower surface of the membrane were fixed with 4% paraformaldehyde and stained with 0.1% crystal violet. The number of infiltrating cells was counted in five randomly selected microscopic fields of each filter.

### Western blotting

HCC tissues and cells were homogenized in SDS sample buffer (10% glycerol, 2% SDS, 0.01% bromophenol blue, 1.25% 2-beta-mercaptoethanol, 25 mM Tris–HCl, pH 6.8) with ULTRA-TURRAX (IKA, Germany) at 4 °C. Protein concentration was determined using the Quick Start™ Bradford protein assay kit (Bio-Rad, USA). Ten micrograms of total protein extracts was loaded in 10% SDS-PAGE and transferred to 0.45-μm PVDF membranes (Millipore, USA) using an electro-blotting apparatus (Bio-Rad, USA). Anti-B3GALNT2, anti-BDH2, and anti-GAPDH antibodies were purchased from Proteintech. The Immobilon™ Western Chemiluminescence HRP substrate kit (Millipore, USA) was used for chemiluminescence. Images were obtained with the ImageQuant™ LAS-4000 (Amersham Biosciences, GE, USA) and quantified using the ImageQuant™ TL software (version 7.0, Amersham Biosciences, GE, USA).

### Quantitative real-time PCR

Total RNA of the samples was purified using TRIzol (Invitrogen, Carbad, CA, USA) and then reverse-transcribed to cDNA using the PrimeScript RT reagent kit (Takara, Tokyo, Japan). Real-time PCR was performed with cDNA production using SYBR Premix Ex Taq (Takara, Tokyo, Japan) on an ABI StepOne Plus (Applied Biosystems, USA) instrument. GAPDH was used as an internal control. Primers used in this study are listed as follows: B3GALNT2 forward: GATGTGGTAGTTGGCGTGTTG, reverse: CGTTGACTTAATGTGGGATGCTG, GAPDH forward: GAGTCAACGGATTTGGTCGT, reverse: TTGATTTTGGAGGGATCTCG, BDH2 forward: GCTTCCA GCGTCAAAGGAGTT, reverse: CAGTTGCGAATCTTCCCGTC, MIF forward: TACACCCAGACCAAATGATG, reverse: TTCTCCTAATGCTCCAATACTG.

### Immunohistochemistry

Immunohistochemistry (IHC) tests on tissue microarray and paraffin sections were performed using a Dako REAL EnVision Detection System (Dako, Denmark) following the protocol recommended, and hematoxylin was used for counterstaining. Anti-B3GALNT2, anti-CD206, anti-F4/80, and anti-CD68 antibody were used to quantify relative expression levels. Immunohistochemical scoring was determined as previously described [18]. The staining intensity was scored as 0 for negative, 1 for weak, 2 for moderate weak, 3 for moderate strong, and 4 for strong. The score for the stained area was set as 0 for 0–33%; 1, 33–66%; and 2, 66–100%. The final staining score was obtained by multiplying the staining intensity score by the staining area score, and the results are a series of numbers ranging from 0 to 8.

### ELISA

MIF, CSF1, CCL2, VEGF, and MIP-1α in the culture supernatants of HCC cell lines were measured using ELISA kits (R&D Systems). The culture supernatants of the cells were collected and centrifuged at 500×*g* for 5 min to remove cellular debris. The ELISA was performed according to the manufacturer’s instructions.

Protein and small molecule components were separated using a 3-kDa ultrafiltration tube purchased from Millipore. Fraction containing small molecules (< 3 kDa) was collected from the residual liquid at the first ultrafiltration. The concentrated fraction (> 3 kDa) was further washed three times using PBS and finally concentrated in PBS.

### MIF tautomerase activity assay

The tautomerase activity of MIF in the medium was detected as previously described with minor modifications [19]. Phenylpyruvate could be conversed from enol- to keto- type by MIF, and this reaction was monitored by the decrease of absorbance at 288 nm on a spectrophotometer at room temperature. The assay mixture contained 50 mM sodium-phosphate buffer (pH 6.5) and a series of diluted MIF-containing culture medium. The assay was initiated by addition of ethanol-diluted phenylpyruvic acid with final concentration of 100 μm. Absorbance values for each group were normalized with the control group that contained buffer and fresh DMEM medium, thus yielding the relative percent of enzyme activity. For acetoacetate addition, 10 μM lithium acetoacetate was used. All the chemicals were purchased from Sigma (St. Louis, MO, USA).

For MIF inhibition, *N*-acetyl-p-benzoquinone (NAPQI) was purchased from Sigma (St. Louis, MO, USA), and it could inhibit 96% of MIF by incubating with cells for 5 min at 200 μM as previously reported [20].

### Animal models

All animal experiments were approved by the research medical ethics committee of Fudan University (Shanghai, China; approval no. 170013-0056) and were performed in accordance with the approved guidelines. Nude mice were purchased from the Shanghai Laboratory Animal Center of Chinese Academy Sciences (Shanghai, China) and were housed in individual ventilated cages. All of the mice were randomly grouped (*n* = 6 in each group).

For the subcutaneous xenograft model, Huh7-luc cells were resuspended in PBS (5 × 10^6^/mouse) and subcutaneously inoculated into the axillaries of 4-week-old male nude mice. The mice were sacrificed after 4 weeks, and tumor tissues were harvested and weighted. For the orthotopic translation model, tumors from xenograft models were separated and chopped in PBS at 4 °C. The diameter of each fragment was modified to 1 mm. The fragments were then transplanted into nude mice in the left lobes of the liver. Bioluminescent imaging was performed with an IVIS200 (Xenogen, Caliper, CA) 10 min after intraperitoneal injection of luciferin (3 mg/mouse) (Promega, WI, USA). The intensity of luciferase signals was quantified using ROI analysis.

### Metabolomics analysis

For metabolomics analysis, 7402-B3GALNT2 and 7402-control cells were cultured to 80% confluence and the medium was removed, followed by washing the cells with ice-cold PBS. The cells were then collected into tubes with PBS by scraping. For targeted metabolomics analysis, the culture medium was changed to serum-free medium 2 h before metabolite collection. Metabolite fractions of the culture media were collected and analyzed by targeted LC-MS/MS. LC-MS analysis was performed as described previously [21].

An ACQUITY UHPLC System (Waters Corporation, Milford, USA) coupled with an AB SCIEX Triple TOF 5600 System (AB SCIEX, Framingham, MA) was used to analyze the metabolic profiling. An ACQUITY UPLC BEH C18 column (1.7 μm, 2.1 × 100 mm) was employed with a binary gradient method. Data acquisition was performed in full scan mode (m/z ranges from 70 to 1000) combined with information-dependent acquisition (IDA) mode. For IDA analysis, range of m/z was set as 50–1000 and the collision energy was 30 eV. The QCs were injected at regular intervals (every eight samples) throughout the analytical run to provide a set of data from which repeatability could be assessed.

The raw data were converted to common data format (mzML) files using a conversion software program MSconventer. Metabolomics data were acquired using software XCMS 1.50.1 version, which produced a matrix of features with the associated retention time, accurate mass, and chromatographic separation. The positive and negative data were combined to get a combined data set which was imported into the SIMCA software package (version 14.0, Umetrics, Umea, Sweden). Principal component analysis (PCA) was carried out to visualize metabolic alterations among experimental groups, after mean centering and unit variance scaling. The differential metabolites were selected on the basis of *p* values from a two-tailed Student’s *t* test on the normalized peak areas, where metabolites with *p* values less than 0.05 were included. A reference material database built by the Dalian Institute of Chemical Physics, Chinese Academy of Sciences and Dalian ChemData Solution Information Technology Co., Ltd., HMDB, and METLIN was used.

### Statistical analysis

All analyses were performed with SPSS 13.0 (Chicago, IL, USA). Results were presented as the mean ± standard deviation with at least three replicates for each sample. Optimal cut-off values for B3GALNT2 expression were determined by ROC curve analysis. Pearson’s chi-square test was used to identify the correlation between B3GALNT2 expression and other factors. Survival probability was determined by the Kalan-Meier curve, and the differences between groups were assessed by Log-rank test. Univariate and multivariate survival analyses were applied using Cox regression. Differences between groups were determined with Student’s *t* test. Statistical significance was set at two tails *p* < 0.05.

## Results

### Upregulation of B3GALNT2 in HCC is associated with poor prognosis

**Fig. 1:**
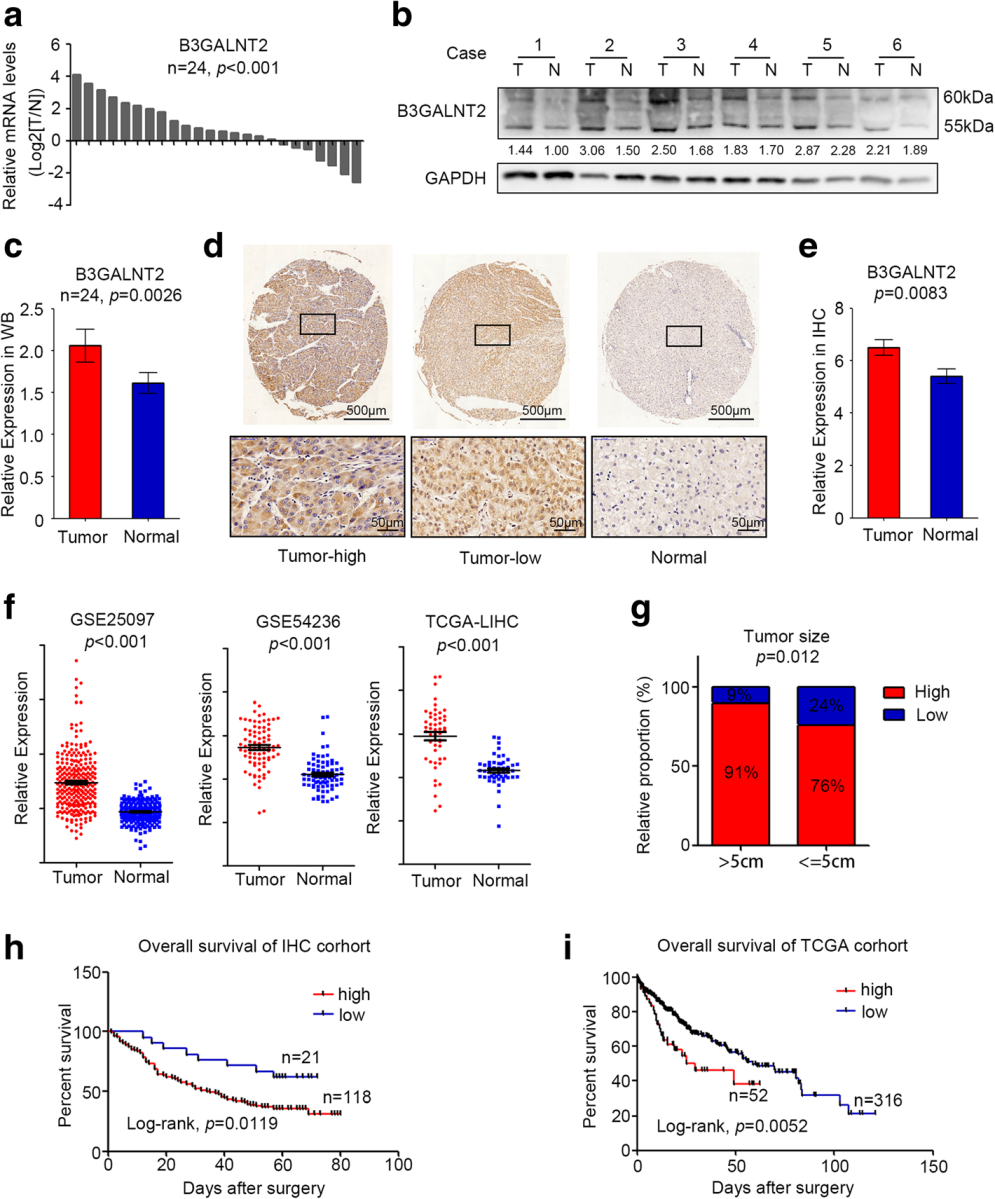
B3GALNT2 is upregulated in hepatocellular carcinoma and correlated with poor prognosis. **a** Relative mRNA expression of B3GALNT2 was determined by real-time PCR in 24 pairs of HCC tumor tissues and adjacent non-tumor tissues. **b** Western blot analysis was used to determine the protein expression level of B3GALNT2 in 24 pairs of HCC tissues. Six representative pairs are shown. T HCC tumor tissue, N adjacent non-tumor tissue. **c** Statistical data of Western blot analysis on HCC tumor tissues and adjacent non-tumor tissues. **d** Representative IHC staining of adjacent non-tumor tissue and tumor tissue with high or low levels of B3GALNT2 in HCC. Regional magnification images are shown below. **e** Statistical data of the IHC staining score. **f** Relative mRNA expression of B3GALNT2 in HCC tumor tissues and normal liver tissues obtained from GSE25097, GSE54236, and TCGA-LIHC datasets. **g** Correlation between B3GALNT2 expression and tumor size in IHC cohort. **h**, **i** Kaplan-Meier analysis for overall survival of HCC patients from IHC cohort and TCGA-LIHC dataset

We first determined the expression pattern of different B3GALTs and B3GALNTs in public HCC datasets and found that B3GALNTs instead of B3GALTs were significantly upregulated in HCC (Additional file 1: Figure S1a–c). Since the upregulation of B3GALNT2 was higher than that of another B3GALNT member (Additional file 1: Figure S1a–c), we focused on the roles of B3GALNT2 in HCC. To verify the expression pattern of B3GALNT2 in HCC, we examined mRNA levels of B3GALNT2 in 24 pairs of samples from HCC tumor tissues and matched adjacent normal tissues. Higher levels of B3GALNT2 in tumor tissues compared with adjacent normal tissues were observed in most samples (*p* < 0.001) (Fig. 1a). In accordance with the mRNA levels, higher protein levels of B3GALNT2 in tumor tissues were also determined by Western blotting analysis (Fig. 1b, c, and Additional file 1: Figure S1d). Similar results were observed in IHC analysis on 139 pairs of HCC samples (*p* = 0.0083) (Fig. 1d, e). Upregulation of B3GALNT2 in HCC was further confirmed by the published HCC datasets from The Cancer Genome Atlas (TCGA) database (TCGA-LIHC) and Gene Expression Omnibus (GEO) datasets (GSE25097 [22] and GSE36376 [23]) (Fig. 1f). These results indicate that B3GALNT2 is upregulated in HCC.

Receiver operating characteristic (ROC) curve analysis grouped all the patients according to B3GALNT2 levels in tumor tissues, and representative IHC staining on B3GALNT2^high^ and B3GALNT2^low^ samples is shown respectively (Fig. 1a, and Additional file 1: Figure S1e). Chi-square test was applied to analyze correlations between intra-tumoral B3GALNT2 levels and clinicopathological features in HCC patients, and the data show that upregulation of B3GALNT2 is significantly correlated with tumor size (*p* = 0.01188) and T stage (*p* = 0.00328) among all the pathological factors (Fig. 1g, Additional file 2: Table S1). Furthermore, Kaplan-Meier analysis on both the IHC cohort and the TCGA cohort revealed that overexpressed B3GALNT2 was significantly associated with poorer overall survival (*p* = 0.0119 for IHC cohort and *p* = 0.0052 for TCGA cohort) (Fig. 1h, i). Meanwhile, univariate and multivariate Cox analysis revealed the prognostic significance of B3GALNT2 expression for overall survival of HCC patients (HR, 0.468; 95% CI, 0.252–0.872; *p* < 0.001) (Additional file 3: Table S2). Taken together, these data indicate that upregulation of B3GALNT2 is closely associated with HCC progression.

### B3GALNT2 in HCC cells confers no significant function in vitro whereas it promotes tumor progression in vivo

**Fig. 2:**
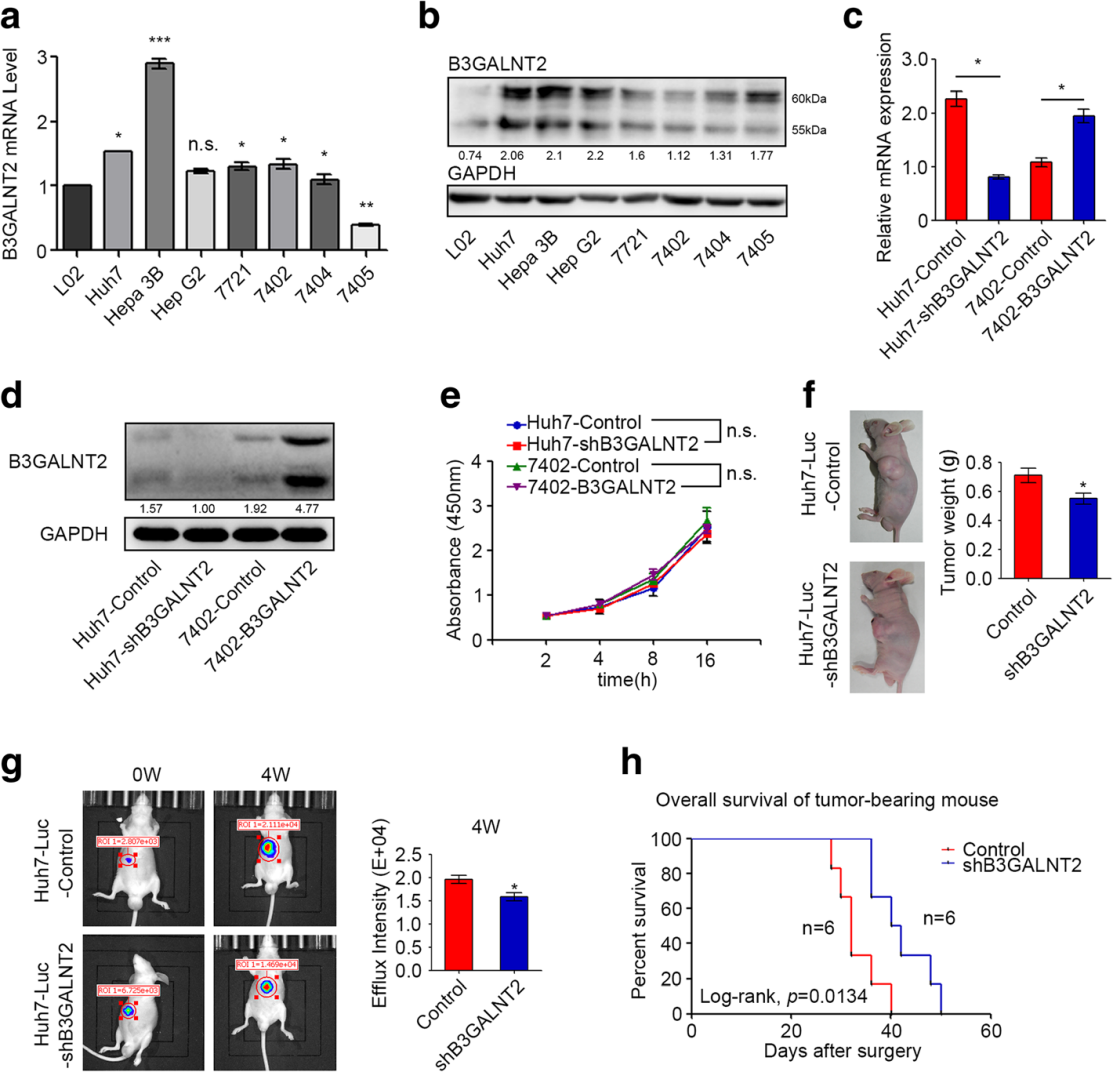
B3GALNT2 confers no effects on HCC cells in vitro whereas it promotes tumor growth in vivo. **a**, **b** The mRNA and protein level of B3GALNT2 in L02 and seven hepatocellular carcinoma cell lines was determined by qPCR and Western blot. **c**, **d** Knockdown and overexpression efficiency of B3GALNT2 in Huh7 and 7402 cell lines was determined by qPCR and Western blot. **e** CCK-8 analysis of Huh7-shB3GALNT2 and 7402-B3GALNT2 cells and their control cells. **f** In vivo effect of B3GALNT2 in subcutaneous xenograft model at 4th week after inoculation (*n* = 6 in each group). Representative photos of tumor-bearing mice are shown on the left, and statistical data is shown on the right. **g** In vivo effect of B3GALNT2 in an orthotopic transplantation model 4 weeks after tumor transplantation (*n* = 6 in each group). Representative images of tumor-bearing mice are shown on the left, and statistical data is shown on the right. **h** Overall survival of orthotopic transplanted mice. *p < 0.05, n.s. not significant

Consistent with the data from HCC patients (Fig. 1), B3GALNT2 was also upregulated in most HCC cell lines compared with the normal liver cell line L02 cells in both mRNA and protein levels (Fig. 2a, b). To further investigate the role of B3GALNT2 in HCC cells, we stably overexpressed B3GALNT2 in BEL-7402 cells that had relatively low levels of B3GALNT2 and Huh7 cells with a relatively high protein level of B3GALNT2. B3GALNT2 stable knockdown was performed followed by Western blot and qRT-PCR to confirm the efficiency of knockdown or overexpression (Fig. 2c, d). Despite the positive correlation between B3GALNT2 and tumor size in clinical pathological analysis (Fig. 1f), no significant effect of B3GALNT2 on cell viability test was observed in CCK8 (Fig. 2e) and neither cell cycle nor apoptosis was influenced by B3GALNT2 knockdown in vitro (Additional file 4: Figure S2a–b). Intriguingly, in the subcutaneous tumor model, B3GALNT2 knockdown significantly suppressed tumor growth (*p* < 0.05) and B3GALNT2 overexpression promoted tumor growth (*p* < 0.05) (Fig. 2f and Additional file 4: Figure S2c). Similar results were observed in liver orthotopic xenograft transplants (*p* < 0.05), and B3GALNT2 knockdown in orthotopic xenografts significantly improved survival of tumor-bearing mice (*p* = 0.0134) (Fig. 2g, h). These data verify the role of upregulated B3GALNT2 in promoting HCC tumor growth. The contradictory results of in vivo and in vitro tests also suggested that other than regulating tumor cells directly, B3GALNT2 might exert its function via modulating some other tumor-associated cells in vivo to promote tumor progression indirectly.

### B3GALNT2 knockdown inhibits macrophage recruitment in HCC cells

**Fig. 3:**
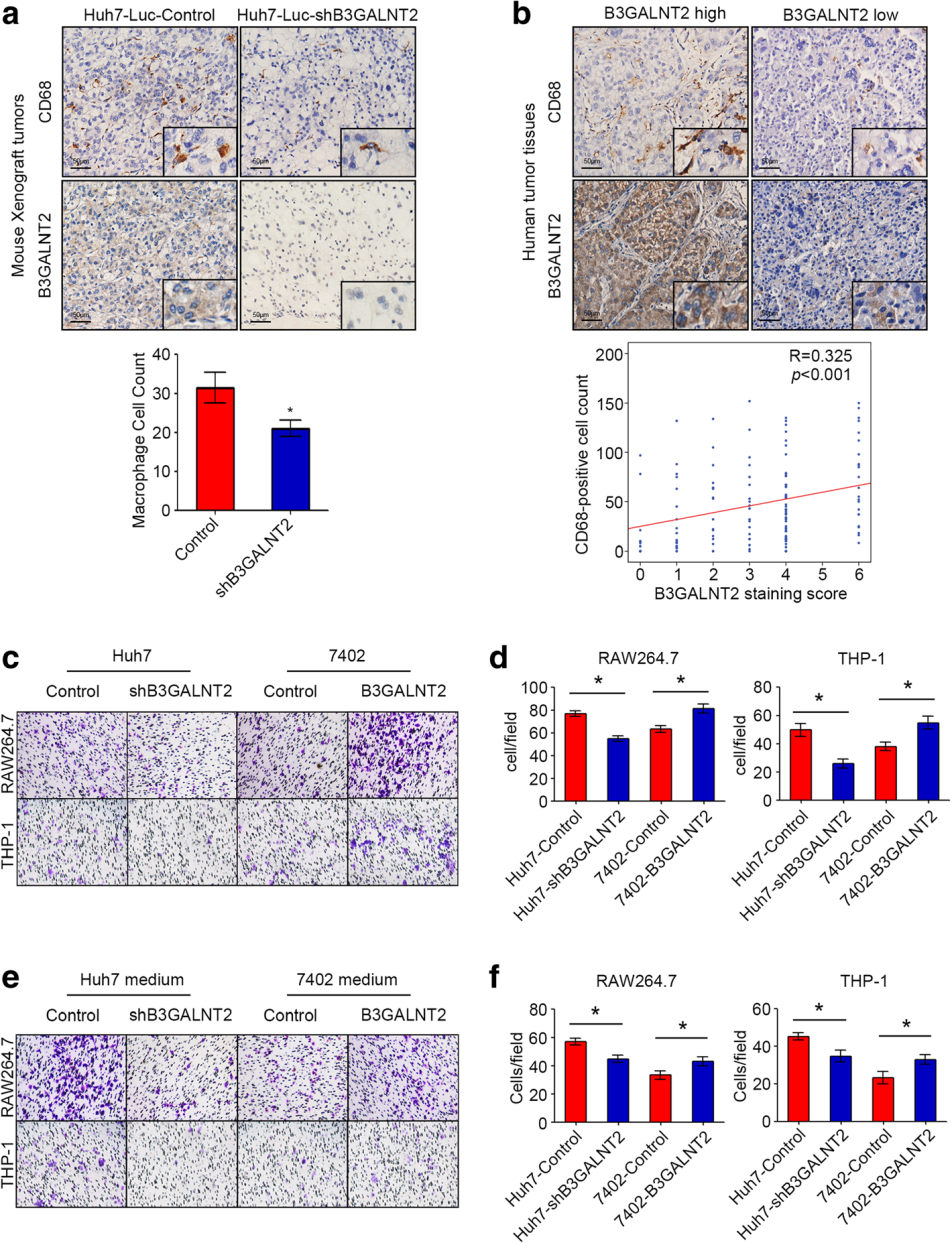
B3GALNT2 knockdown inhibits macrophage recruitment in HCC cells. **a** IHC analysis of B3GALNT2 and F4/80 levels in tumor tissues from orthotopic xenografts. Cell count of macrophages is shown below. **b** Representative IHC staining of B3GALNT2 and CD68 in human HCC tumor tissues. The correlation between B3GALNT2 and CD68 levels in the IHC cohort are shown below. **c**, **d** Macrophage recruitment assessment in transwell assays using HCC cell lines with B3GALNT2 overexpression or knockdown. Both representative transwell images (**c**) and statistical data (**d**) are shown. **e**, **f** Macrophage recruitment assessment in transwell assays using culture medium of HCC cells with B3GALNT2 overexpression or knockdown. Both the representative transwell images (**e**) and statistical data (**f**) are shown. *p < 0.05

In HCC, myeloid cells, especially macrophages, are abundant in the tumor microenvironment and have been linked to uncontrolled malignant growth [24, 25]. To address whether macrophages are involved in the function of B3GALNT2, we examined macrophage numbers in the orthotopic xenograft with or without B3GALNT2 knockdown and found that B3GALNT2 knockdown significantly decreased infiltrated macrophages in the mouse xenograft and B3GALNT2 overexpression conferred opposite effects (Fig. 3a and Additional file 4: Figure S2d). Similar results were observed in tumor tissues from HCC patients and revealed a significant positive correlation between tumor B3GALNT2 levels and the number of CD68-positive macrophages in the HCC microenvironment (*R* = 0.325, *p* < 0.001) (Fig. 3b). Moreover, B3GALNT2 upregulation was also significantly associated with the increase of CD206-positive TAMs (*R* = 0.286, *p* < 0.001) (Additional file 4: Figure S2e–f). These data suggest that B3GALNT2 is involved in the recruitment of macrophages, especially TAMs. To verify the effects of B3GALNT2 on macrophage recruitment, we put HCC cells with different B3GALNT2 levels in the lower chamber and performed in vitro transwell assays for PMA-differentiated human THP1 macrophages and mouse RAW264.7 cells. We found that B3GALNT2 knockdown in Huh7 cells significantly inhibited macrophage infiltration, and overexpressed B3GALNT2 in 7402 cells increased infiltrated macrophages (Fig. 3c, d). Meanwhile, B3GALNT2 promoted recruitment of both mouse and human macrophages. Since HCC tumor cells and macrophages localized in different chambers in the transwell assays, we speculated that B3GALNT2 might modulate the secretion from tumor cells to promote macrophage infiltration. Thus, the conditioned culture medium of HCC cells with B3GALNT2 level changes was collected and added into the lower chambers in the macrophage infiltration assays. The media functioned similarly to that of the secreting host, by proving that B3GALNT2 can promote macrophage infiltration via altering secretions from tumor cells (Fig. 3e, f).

### Acetoacetate is identified as the key secreted molecule from HCC cells in B3GALNT2-mediated macrophage recruitment

**Fig. 4:**
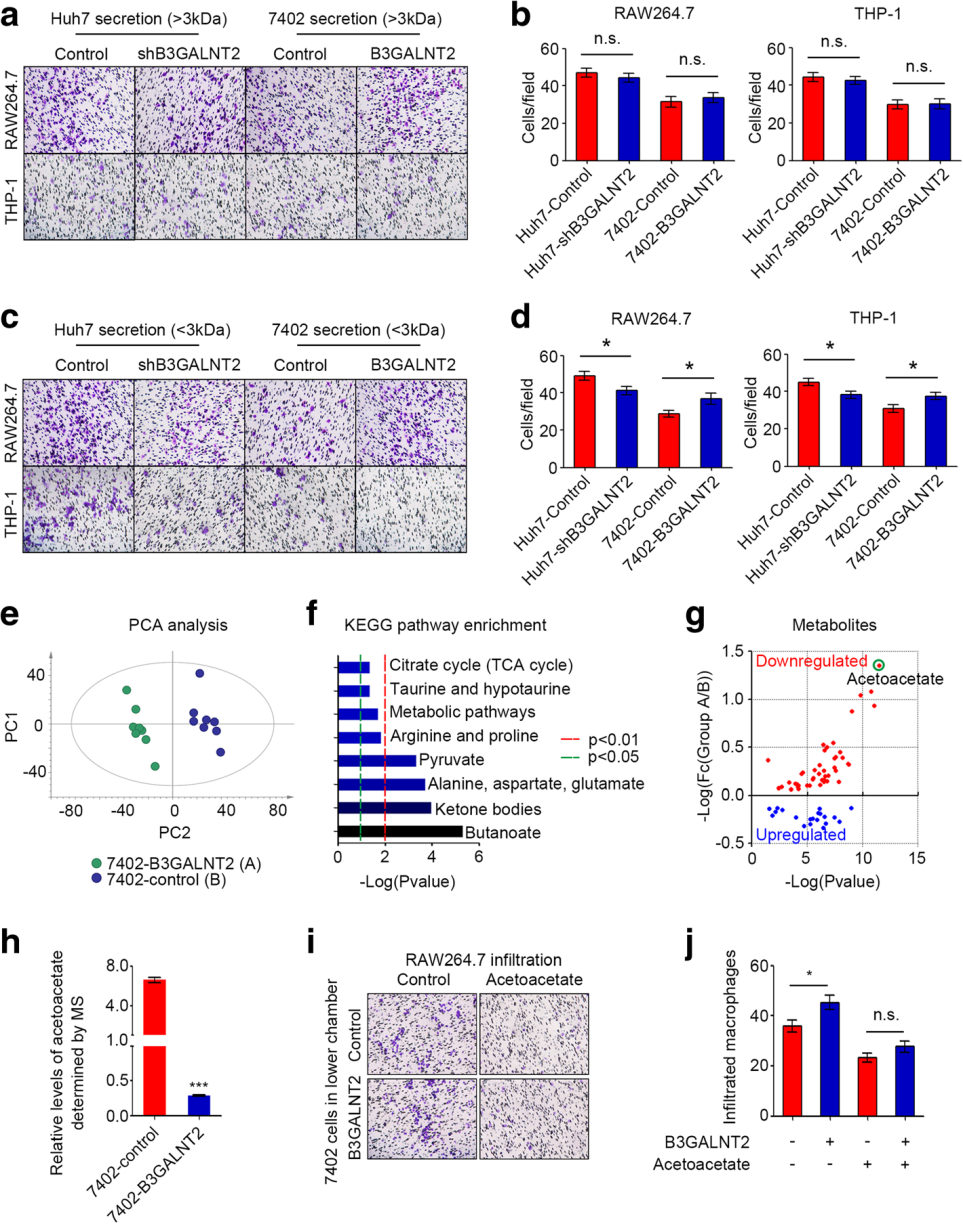
Acetoacetate is identified as the key secreted molecule from HCC cells in B3GALNT2-mediated macrophage recruitment. **a**, **b** Macrophage recruitment assessment in transwell assays using large-molecule fraction (> 3 kDa) of the culture medium from HCC cells with B3GALNT2 overexpression or knockdown. **c**, **d** Macrophage recruitment assessment in transwell assays using the small-molecule fraction (< 3 kDa) of the culture medium from HCC cells with B3GALNT2 overexpression or knockdown. **e** PCA analysis for the metabolite profiles of B3GALNT2-overexpressed 7402 cells and control cells determined by LC-MS. **f** KEGG pathway enrichment for the metabolites significantly changed by B3GALNT2. **g** The metabolite profile changed by B3GALNT2. **h** Relative levels of acetoacetate in 7402-B3GALNT2 cells and 7402-control cells determined by metabolomics analysis. **i**, **j** Macrophage recruitment assessment in transwell assays using 7402 cells with or without B3GALNT2 overexpression in addition with acetoacetate. *p < 0.05, ***p < 0.001, n.s. not significant

To determine key factors in B3GALNT2-associated secretion, we first applied ELISA analysis on some well-studied cytokines that have been reported to promote macrophage recruitment, like CSF1, CCL2, VEGF, and MIP-1α [26, 27]. However, no significant changes of these cytokines were observed in the supernatant when B3GALNT2 was overexpressed or knocked down in HCC cells (Additional file 5: Figure S3). We next concentrated the secretion with a 3-kDa filter. The concentrated secretion from B3GALNT2-overexpressed HCC cells also failed to change the infiltration of macrophages, and similar results were observed when using the concentrated secretion from B3GALNT2-knockdown HCC cells (Fig. 4a, b). Intriguingly, the remaining supernatant that contained molecules smaller than 3 kDa exerted B3GALNT2-mediated functions on macrophage recruitment (Fig. 4c, d). These data indicate that key factors in B3GALNT2-associated functional secretions are small molecules.

Since most metabolites were smaller than 3 kDa, we speculated that the key factor might be a metabolite. We performed metabolomics analysis on B3GALNT2-overexpressed 7402 cells and 7402 parental cells. The total ion chromatogram (TIC) and principal component analysis (PCA) (eight samples in each group) revealed that overexpression of B3GALNT2 altered the metabolomic pattern in 7402 cells (Fig. 4e and Additional file 6: Figure S4). Levels of several metabolites (*n* = 66) were significantly changed by B3GALNT2 (Additional file 7: Table S3), and the functional enrichment analysis of KEGG pathways on these metabolites revealed eight significantly enriched metabolic pathways, including the metabolism of butanoate (*n* = 6, *p* = 4.87E−06); ketone bodies (*n* = 3, *p* = 1.13E−04); alanine, aspartate, and glutamate (*n* = 4, *p* = 1.98E−04); pyruvate (*n* = 4, *p* = 4.87E−04); arginine and proline (*n* = 4, *p* = 1.55E−02); taurine and hypotaurine (*n* = 2, *p* = 0.045); and citrate cycle (*n* = 2, *p* = 0.045) (Fig. 4f and Additional file 7: Table S3). Most (68.2%, 45/66) of these metabolites were significantly downregulated by B3GALNT2 (*p* < 0.05), and acetoacetate conferred the lowest *p* value (*p* = 3.00E−12) and the largest fold change (FC = 22.64) among all downregulated metabolites (Fig. 4g, h, and Additional file 7: Table S3), suggesting that acetoacetate and its related metabolic pathways might be critical for functions of B3GALNT2 in macrophage recruitment. To verify this, we added acetoacetate into the lower chamber containing 7402 cells and found that the overexpression of B3GALNT2 in HCC cells failed to promote macrophage infiltration in addition with acetoacetate (Fig. 4i, j), proving that acetoacetate attenuates the effects of B3GALNT2. Taken together, our data indicate that B3GALNT2 facilitates macrophage recruitment via downregulating acetoacetate levels.

### B3GALNT2 regulates the transcription of some enzymes involved in acetoacetate-related metabolism

**Fig. 5:**
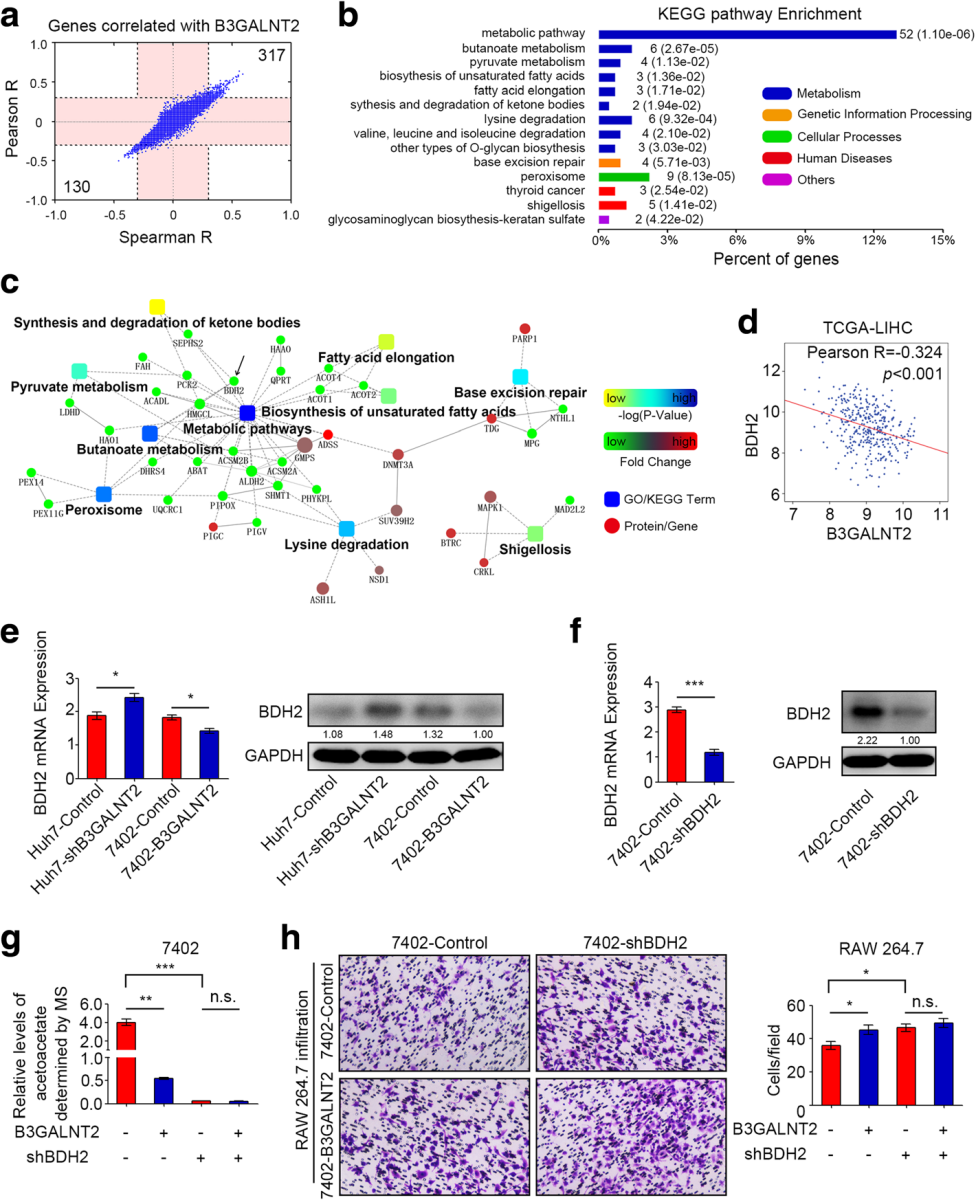
B3GALNT2 regulates the transcription of enzymes involved in acetoacetate-related metabolism. **a** The correlations of all genes from TCGA-LIHC dataset with B3GALNT2. **b** KEGG pathway enrichment for all the genes conferring strong correlation with B3GALNT2 (Spearman |*R*| > 0.3 and Pearson |*R*| > 0.3). **c** The co-expression network of B3GALNT2 based on correlated genes from TCGA-LIHC database. **d** Correlation between B3GALNT2 and BDH2 expression in TCGA-LIHC dataset. **e** Protein and mRNA levels of BDH2 in HCC cells with B3GALNT2 overexpression or knockdown. **f** Knockdown efficiency of BDH2 in 7402 cell lines determined by real-time PCR and Western blot. **g** Secreted acetoacetate levels from 7402 cells with BDH2 knockdown and/or B3GALNT2 overexpression determined by targeted metabolomic analysis. **h** Macrophage recruitment assessment in transwell assays using 7402 cells with BDH2 knockdown and/or B3GALNT2 overexpression. *p < 0.05, **p < 0.01, ***p < 0.001, n.s. not significant

To further investigate the mechanism of how B3GALNT2 downregulates acetoacetate levels, we analyzed all the genes strongly correlated with B3GALNT2 from TCGA data (Pearson |*R*| > 0.3 and Spearman |*R*| > 0.3). We screened out 447 genes that conferred strong correlation with B3GALNT2 (Fig. 5a and Additional file 8: Table S4). Most of the genes (292/447) were metabolic enzymes and were significantly associated with metabolic processes in functional enrichments of both the KEGG pathway (*n* = 52, *p* = 1.10E−06) and Gene Ontology (*n* = 292, *p* = 6.42E−25) (Fig. 5b, Additional file 8: Table S4 and Additional file 9: Figure S5). Some of the enriched metabolic pathways were acetoacetate-related, including the metabolism of butanoate (*n* = 6, *p* = 2.67E−05); lysine (*n* = 6, *p* = 9.32E−04), valine, leucine, and isoleucine (*n* = 4, *p* = 2.10E−02); and ketone bodies (*n* = 2, *p* = 1.94E−02) (Fig. 5b, Additional file 8: Table S4 and Additional file 9: Figure S5). Since BDH2, which directly catalyzes (R)-3-Hydroxybutanoate to acetoacetate, was involved in these metabolic pathways and conferred significant correlation with B3GALNT2 (Pearson *R* = − 0.324, *p* < 0.001), we further determined the relationship with B3GALNT2 and BDH2 in vitro (Fig. 5c, d and Additional file 10: Figure S6). Our data verified that at both mRNA and protein levels, B3GALNT2 negatively regulated BDH2 levels in Huh7 and 7402 cells (Fig. 5e). Meanwhile, BDH2 knockdown in 7402 cells decreased acetoacetate levels and enhanced macrophage infiltration (Fig. 5f–h). Moreover, knockdown of BDH2 attenuated the effects of overexpressed B3GALNT2 on both acetoacetate secretion and macrophage recruitment (Fig. 5f–h). Taken together, these data indicate that B3GALNT2 modulates acetoacetate secretion by regulating expression of acetoacetate-related metabolic enzymes.

### HCC cell-derived acetoacetate suppresses macrophage recruitment by inhibiting MIF activity

**Fig. 6:**
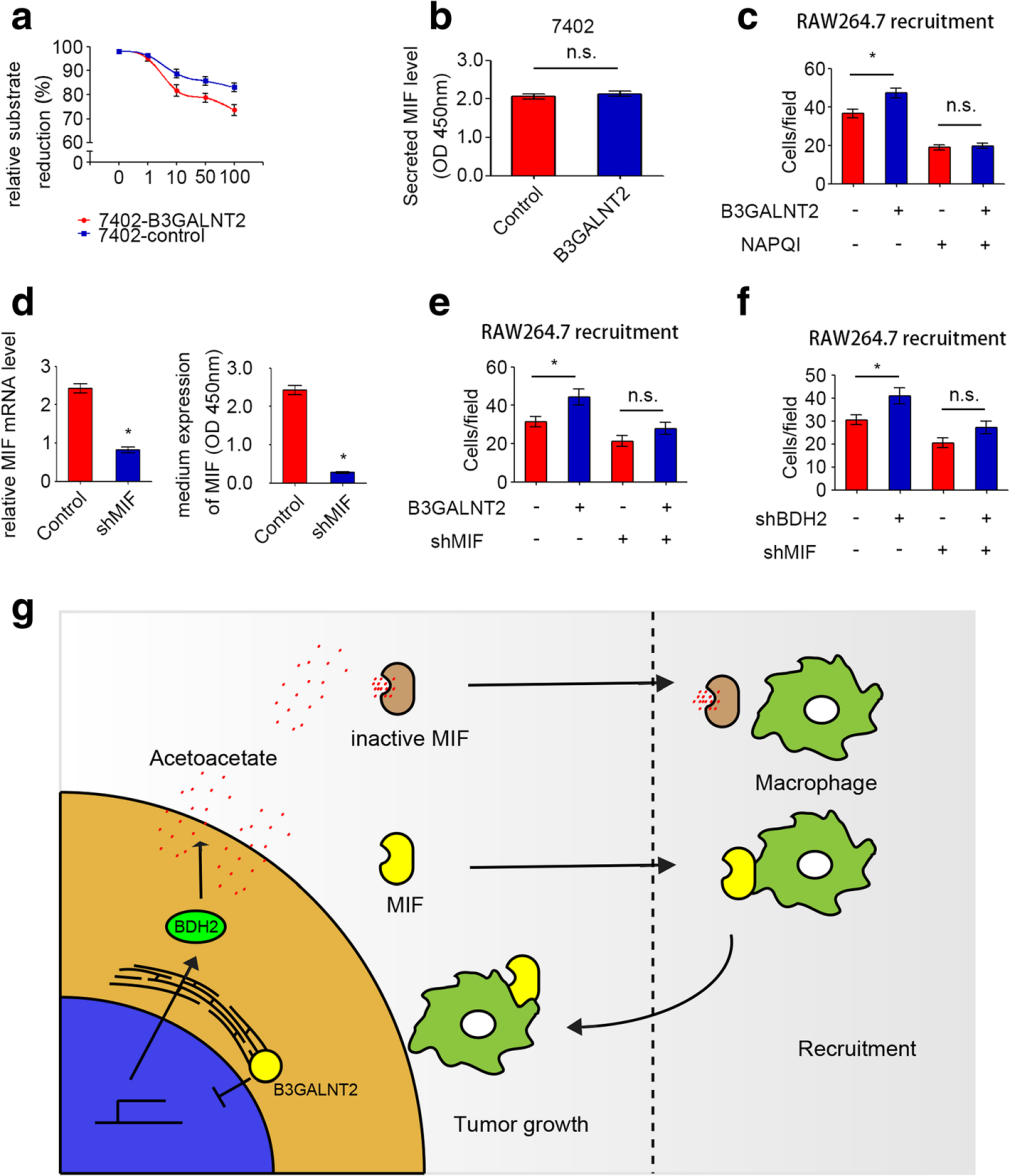
HCC cell-derived acetoacetate suppresses macrophage recruitment by inhibiting MIF activity. **a** The tautomerase activity of secreted MIF from 7402 cells with B3GALNT2 level changes. **b** Secreted levels of MIF in the culture media of B3GALNT2-overexpressed 7402 cells determined by ELISA. **c** NAPQI attenuated the macrophage recruitment ability of B3GALNT2-overexpressed 7402 cells in transwell assays. **d** Knockdown efficiency of MIF in the 7402 cell line as determined by qPCR and ELISA. **e** MIF knockdown attenuated the macrophage recruitment ability of B3GALNT2-overexpressed 7402 cells in transwell assays. **f** MIF knockdown attenuated the macrophage recruitment ability of BDH2-knockdown 7402 cells in transwell assays. **g** Schematic describing how B3GALNT2 functions in HCC

Macrophage migration inhibitory factor (MIF) is a pro-inflammatory cytokine with tautomerase activity and is usually released to the extracellular matrix [28, 29]. Despite its inhibition on random migration, secreted MIF has been reported to promote macrophage recruitment [30]. The tautomerase activity of MIF is associated with its function [31, 32], and ketones like acetoacetate could inhibit the tautomerase activity of MIF [19]. Since tumor B3GALNT2 decreased acetoacetate levels as mentioned above (Fig. 4), we speculated that B3GALNT2-modulated effects on macrophage recruitment are associated with MIF activities. The MIF tautomerase activity assay revealed that B3GALNT2 overexpression elevated the activity of secreted MIF and did not influence MIF levels at the same time (Fig. 6a, b). *N*-acetyl-p-benzoquinone (NAPQI), an inhibitor that specifically suppresses the tautomerase activity of MIF [20], attenuated promotion of B3GALNT2 on macrophage recruitment (Fig. 6c). MIF knockdown in HCC cells also conferred similar effects (Fig. 6d, e). Meanwhile, since BDH2 was one of the enzymes required for B3GALNT2-modulated acetoacetate secretion, we also determined the influence of MIF knockdown on BDH2. Our data show that MIF knockdown attenuates the significant promotion caused by BDH2 knockdown (Fig. 6f). These results indicate that B3GALNT2 is involved in regulating the activity of MIF, which is critical for the function of B3GALNT2 in HCC.

## Discussion

In this study, our data reveal that B3GALNT2 is upregulated in HCC, and this upregulation is associated with tumor growth and poor prognosis. Mechanistically, B3GALNT2 reduced the expression of some metabolic enzymes and thus downregulated the secretion of acetoacetate levels, which relieved the activity of MIF and enhanced macrophage recruitment. Finally, recruited macrophages promoted tumor growth.

HCC is closely related with inflammation. A chronic inflammatory state is required for initiation, and the development of HCC and tumor cells also promote the construction and assist with the maintenance of the inflammatory extracellular environment [24, 33]. Consistent with this, our results prove that HCC cells and their inflammatory microenvironment are mutually beneficial for each other. HCC cells recruit macrophages to maintain the inflammatory environment, and recruited macrophages promote tumor growth in return. Notably, continuous recruitment of inflammatory cells is commonly observed in inflammatory environment. Among these cells, tumor-associated macrophages (TAMs) occupy a major fraction, especially in HCC [24, 25]. Liver-resident Kupffer cells and TAM in HCC are also polarized from peripheral blood mononuclear cell (PMBC), for which monocyte recruitment is indispensable [34].

Monocyte recruitment depends on some cytokines, such as chemokine (C–C motif) ligand 2 (CCL2), macrophage colony-stimulating factor (M-CSF), macrophage inflammatory protein 1a (MIP-1a), vascular endothelial growth factor (VEGF), CCL4, CCL5, CCL8, angiopoietin-2, and MIF [26, 27]. Usually, tumor cells elevate the secretion of cytokines to promote TAM recruitment. In our study, instead of increasing cytokine levels directly, B3GALNT2 elevated the activity of one cytokine, MIF. Although MIF was first identified as an inhibitor of macrophage migration [35], later studies revealed that it has pleiotropic effects on cell migration and chemotaxis [36, 37]. Actually, MIF can induce macrophage recruitment through CCL2 and its receptor CCR2 [30]. Sometimes, MIF acts as a counter-regulation factor against anti-inflammatory and immunosuppressive machinery by overriding the glucocorticoid inhibition to immune response like T cell proliferation and cytokine production [38, 39]. MIF has also been identified as a key cytokine for TAM polarization in melanoma-bearing mice. MIF deficiency or treatment with a MIF antagonist attenuated tumor-induced TAM polarization and reduced expression of angiogenesis-related genes in TAMs [40].

Different from most cytokines, MIF exhibits perplexing tautomerase activity which is conversion of d-dopachrome and phenylpyruvate [41], but its natural substrate is still not clear. Early studies considered that the tautomerase active site is vestigial with no true physiological function [42]. But some researchers revealed that MIF interplays with CD74 as a cytokine and initiates signaling [43, 44]. This interplay could be disrupted when the tautomerase pocket of MIF is mutated or occupied by some molecules [31, 32]. NAPQI that we used in this study is one of the inhibitors targeting the tautomerase pocket of MIF. Our data verify that suppressing the tautomerase activity of MIF attenuates its promotion of macrophage recruitment.

Some natural small molecules, like ketone bodies, also show the ability to inhibit tautomerase activity of MIF [19]. As one of the ketone bodies, acetoacetate inhibited MIF activity in our study and we proved that acetoacetate was the key molecule by which B3GALNT2 regulated MIF activity and macrophage recruitment. Although acetoacetate was reported to promote tumor growth in melanoma [45, 46], ketone bodies including acetoacetate inhibit tumor progression in other cancers [47–50]. It is reported that cancer cell lines grown in glucose plus acetoacetate medium show tightly coupled reduction of growth and ATP concentration [50]. And an acetoacetate-related ketogenic diet decreases tumor cell viability and prolongs survival of mice with metastatic cancer [48]. Meanwhile, since ketone bodies could serve as energy sources in tumor cells, the consumption of these ketone bodies might result in their decrease in extracellular microenvironment. In liver cancers, the consumption of acetoacetate was elevated for lipogenesis to compensate the energy from truncated TCA cycle [51, 52]. Our data suggested that this persistent consumption decreases the extracellular acetoacetate levels and preserves an acetoacetate-low microenvironment benefit for tumor development. Our data also indicated that besides the direct inhibition on cell proliferation, acetoacetate could suppress tumor growth via inhibiting TAM recruitment.

The chronic inflammatory microenvironment is required for both tumor initiation and tumor progression, and increased cytokine secretion in the tumor microenvironment promotes recruitment of immune cells including TAMs [24, 25]. TAMs play important roles in different cancers including HCC [24, 33]. Targeting TAMs is becoming a promising strategy in treating tumors. Some studies suggest that suppressing TAM recruitment via targeting chemokines could also inhibit tumor progression. However, blocking chemokines directly with antibodies sometimes leads to unpredictable tumor growth and distant metastasis. Here, our study provides a new approach to treat HCC by increasing the small molecule acetoacetate. Due to its endogenous derivation, this strategy might be safer and have more efficiency.

## Conclusions

In summary, this study evaluated B3GALNT2 as a tumor marker in HCC and revealed the role of B3GALNT2 in metabolism which transformed the microenvironment of HCC. Our mechanistic study also emphasizes critical roles for acetoacetate and macrophages in HCC tumor growth. Therefore, this study provides more evidence for an advantage of a ketogenic diet to treat HCC and suggests an approach for immunotherapeutic treatment of HCC. Further studies on the molecular mechanism of how B3GALNT2 regulates acetoacetate-related enzymes in HCC progression are required.

## Additional files

Additional file 1: **Figure S1.** B3GALNT2 levels determined by W.B. and ROC curve. a–c Relative mRNA expression of B3GALNT2 in HCC tumor tissues and normal liver tissues obtained from GSE76427, GSE36376, and TCGA-LIHC datasets. d Western blot analysis of B3GALNT2 levels in 24 pairs of HCC tissues. T HCC tumor tissue, N adjacent non-tumor tissue. e ROC curve analysis of the sensitivity and specificity for the predictive value of TNM model, B3GALNT2 expression, and the combination model. (TIFF 546 kb)

Additional file 2: **Table S1.** Relationships between the B3GALNT2expression and the clinicopathological variables of hepatocellular carcinoma patients. (DOCX 20 kb)

Additional file 3: **Table S2.** Univariate and multivariate Cox regression analysis for overall survival of hepatocellular carcinoma patients. (DOCX 20 kb)

Additional file 4: **Figure S2.** Different effects of B3GALNT2 in vitro and in vivo. a Cell cycle analysis of Huh7-shB3GALNT2 and control cells. b Annexin V and PI staining of Huh7-shB3GALNT2 and control cells which show the effects of B3GALNT2 on apoptosis. c In vivo effect of B3GALNT2 overexpression in subcutaneous xenograft model at 4th week after inoculation (*n* = 6 in each group). Representative photos of tumor-bearing mice are shown on the left, and statistical data is shown on the right. d IHC analysis of B3GALNT2 and F4/80 levels in tumor tissues from xenografts. Cell count of macrophages is shown on the right. e Representative IHC staining of B3GALNT2, CD68, and CD206 in human HCC tumor tissues. f Correlation between B3GALNT2 and CD206 levels in human HCC cohort. (TIFF 5233 kb)

Additional file 5: **Figure S3.** Secreted levels of some cytokines. Secreted levels of macrophage-recruiting cytokines in the culture media of B3GALNT2 overexpression or knock down HCC cells as determined by ELISA. (TIFF 153 kb)

Additional file 6: **Figure S4.** The total ion chromatogram for metabolomics analysis. a The total ion chromatogram (TIC) of 7402-B3GALNT2 and 7402-control in negative ion mode. b The total ion chromatogram (TIC) of 7402-B3GALNT2 and 7402-control in positive ion mode. (TIFF 574 kb)

Additional file 7: **Table S3.** B3GALNT2-associated metabolites determined by LC-MS and the functional enrichment of KEGG pathway on these metabolites. (XLSX 39 kb)

Additional file 8: **Table S4**. Genes correlated with B3GALNT2 from TCGA data and their Functional enrichments. (XLSX 29 kb)

Additional file 9: **Figure S5.** Gene Ontology analysis of genes correlated with B3GALNT2. a Biological process enrichment in the GO analysis of genes correlated with B3GALNT2 in TCGA-LIHC dataset. b Molecular function enrichment in the GO analysis of genes correlated with B3GALNT2 in the TCGA-LIHC dataset. (TIFF 322 kb)

Additional file 10: **Figure S6.** KEGG pathway schematic. KEGG pathway schematic of butanoate metabolism marked with the enzymes and metabolites correlated with B3GALNT2. Blue is for enzymes, red is for metabolites. (TIFF 82 kb)
