# Supplementary figures and images for: Increased B3GALNT2 in hepatocellular carcinoma promotes macrophage recruitment via reducing acetoacetate secretion and elevating MIF activity
Source: J Hematol Oncol. 2018 Apr 4;11:50. doi: 10.1186/s13045-018-0595-3 (PMC5885466; doi:10.1186/s13045-018-0595-3)

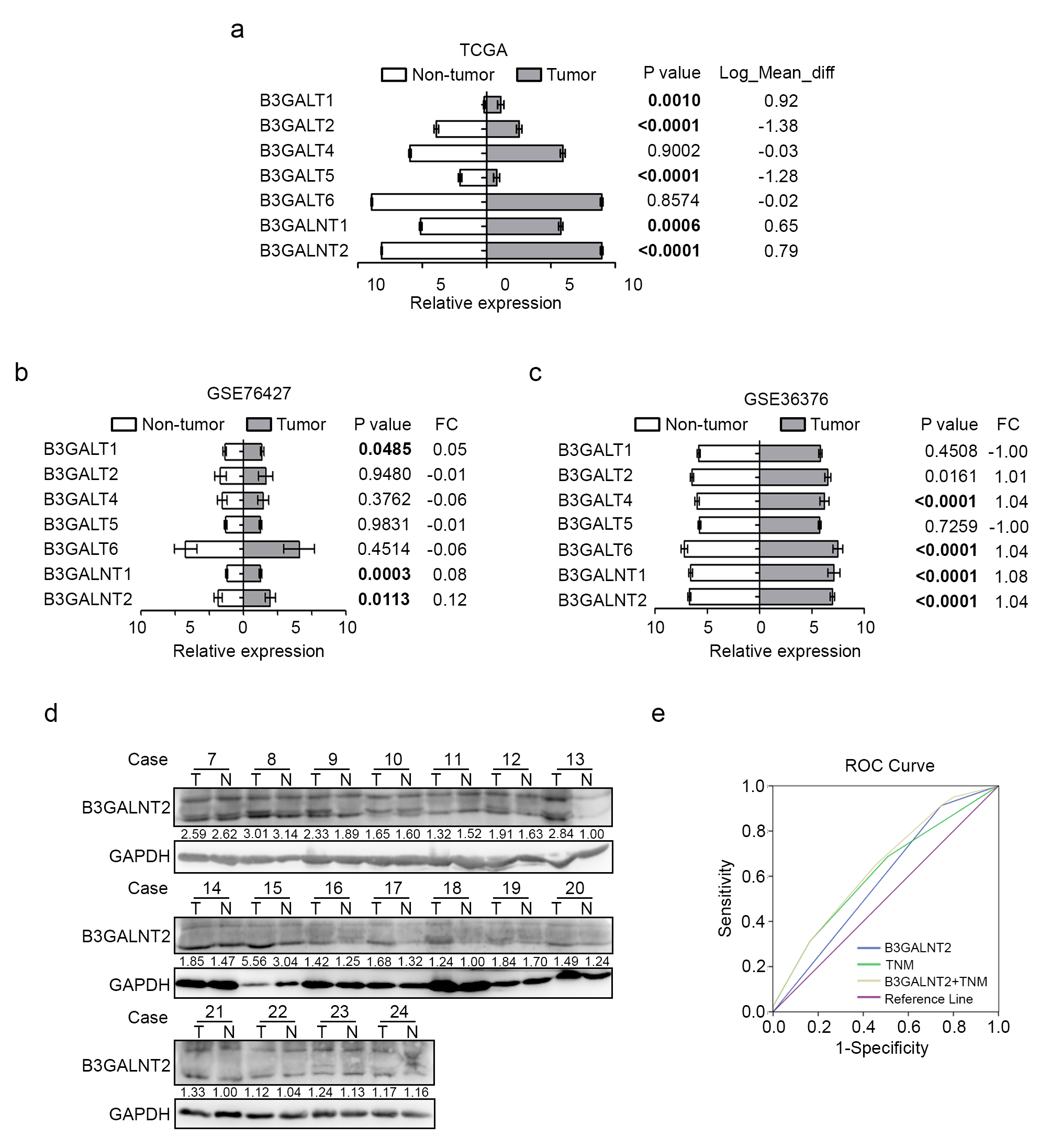

Supplement: Supplementary file 1 — Figure S1. B3GALNT2 levels determined by W.B. and ROC curve. a–c Relative mRNA expression of B3GALNT2 in HCC tumor tissues and normal liver tissues obtained from GSE76427, GSE36376, and TCGA-LIHC datasets. d Western blot analysis of B3GALNT2 levels in 24 pairs of HCC tissues. T HCC tumor tissue, N adjacent non-tumor tissue. e ROC curve analysis of the sensitivity and specificity for the predictive value of TNM model, B3GALNT2 expression, and the combination model. (TIFF 546 kb) [file 13045_2018_595_MOESM1_ESM.tif]

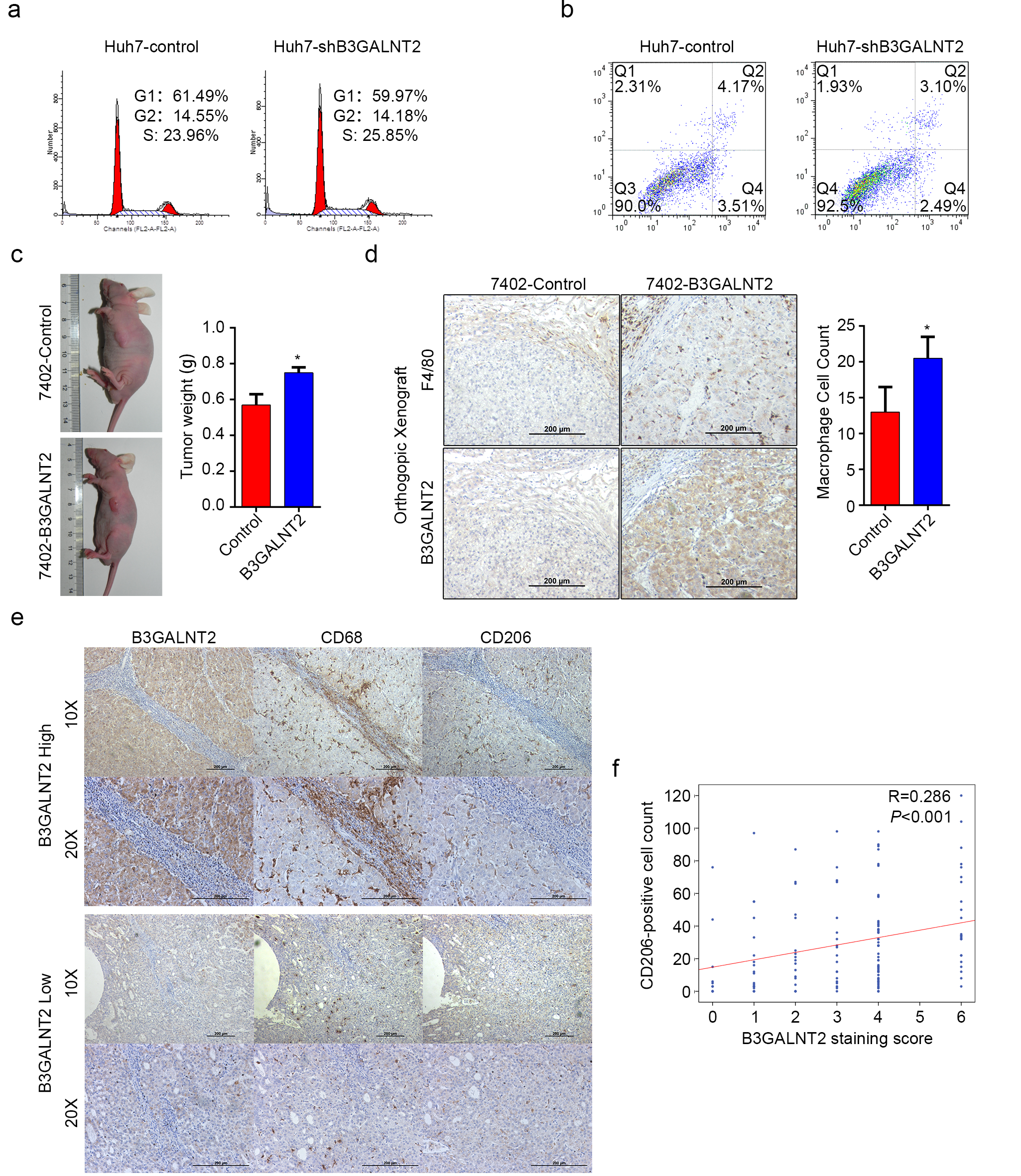

Supplement: Supplementary file 4 — Figure S2. Different effects of B3GALNT2 in vitro and in vivo. a Cell cycle analysis of Huh7-shB3GALNT2 and control cells. b Annexin V and PI staining of Huh7-shB3GALNT2 and control cells which show the effects of B3GALNT2 on apoptosis. c In vivo effect of B3GALNT2 overexpression in subcutaneous xenograft model at 4th week after inoculation (n = 6 in each group). Representative photos of tumor-bearing mice are shown on the left, and statistical data is shown on the right. d IHC analysis of B3GALNT2 and F4/80 levels in tumor tissues from xenografts. Cell count of macrophages is shown on the right. e Representative IHC staining of B3GALNT2, CD68, and CD206 in human HCC tumor tissues. f Correlation between B3GALNT2 and CD206 levels in human HCC cohort. (TIFF 5233 kb) [file 13045_2018_595_MOESM4_ESM.tif]

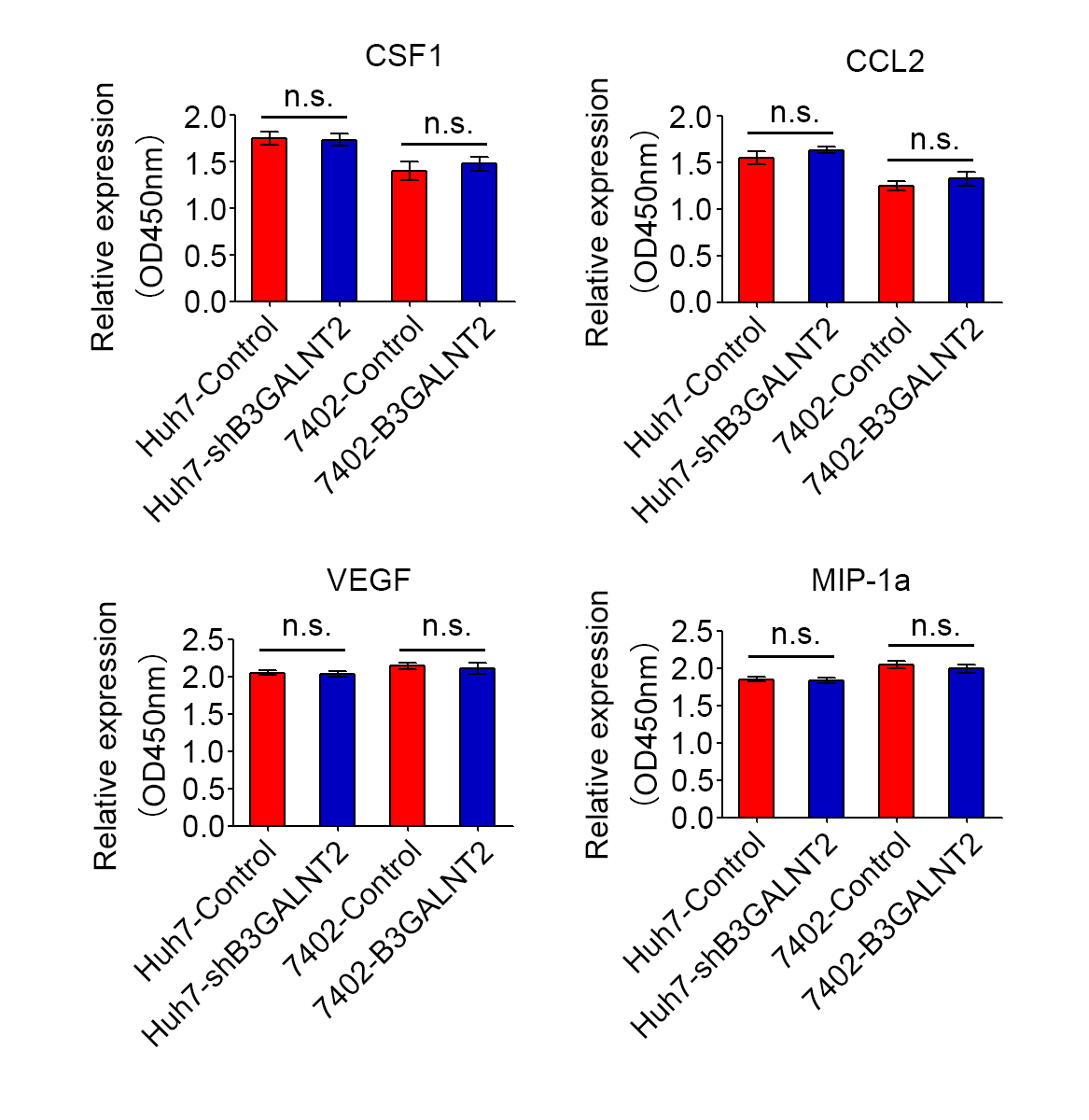

Supplement: Supplementary file 5 — Figure S3. Secreted levels of some cytokines. Secreted levels of macrophage-recruiting cytokines in the culture media of B3GALNT2 overexpression or knock down HCC cells as determined by ELISA. (TIFF 153 kb) [file 13045_2018_595_MOESM5_ESM.tif]

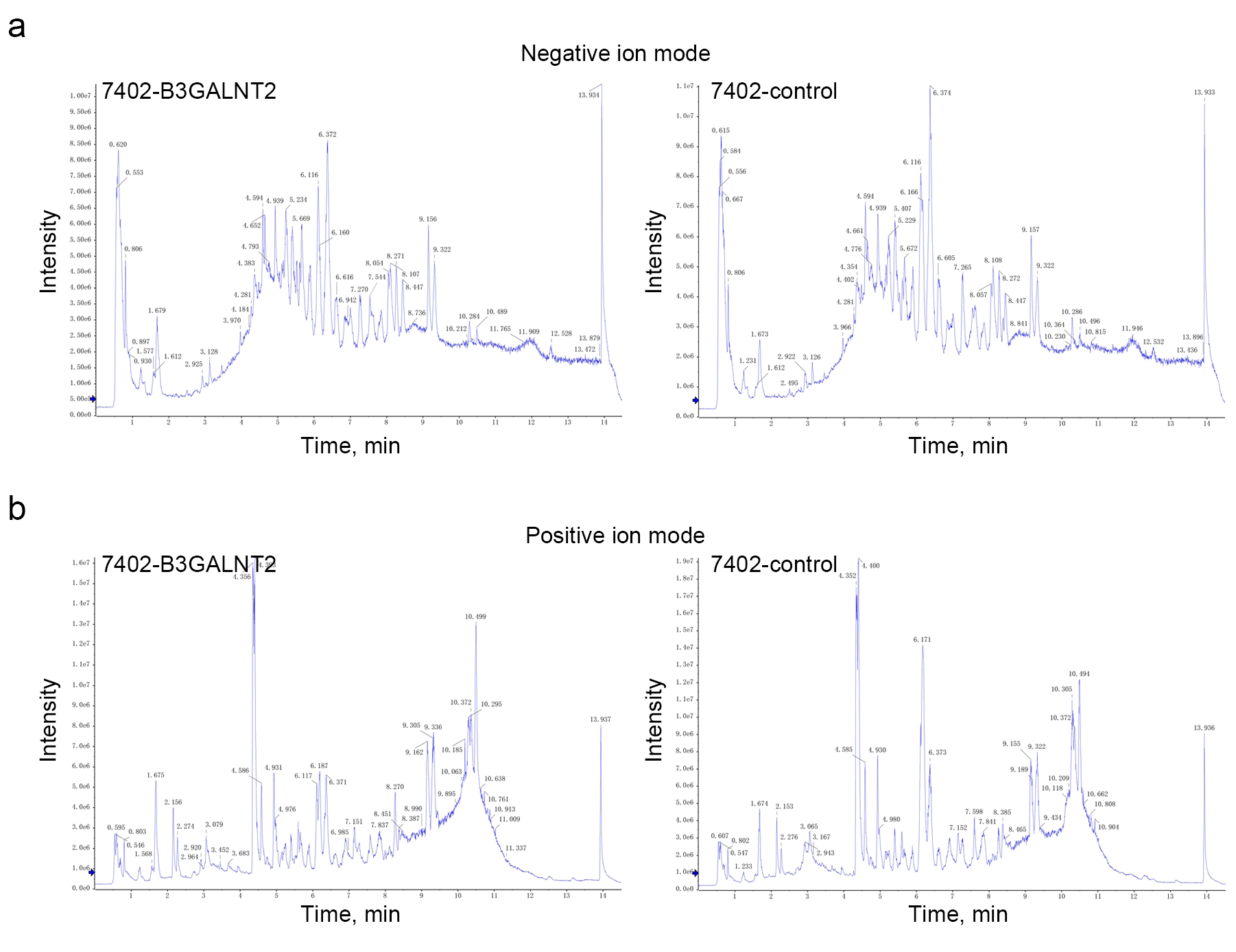

Supplement: Supplementary file 6 — Figure S4. The total ion chromatogram for metabolomics analysis. a The total ion chromatogram (TIC) of 7402-B3GALNT2 and 7402-control in negative ion mode. b The total ion chromatogram (TIC) of 7402-B3GALNT2 and 7402-control in positive ion mode. (TIFF 574 kb) [file 13045_2018_595_MOESM6_ESM.tif]

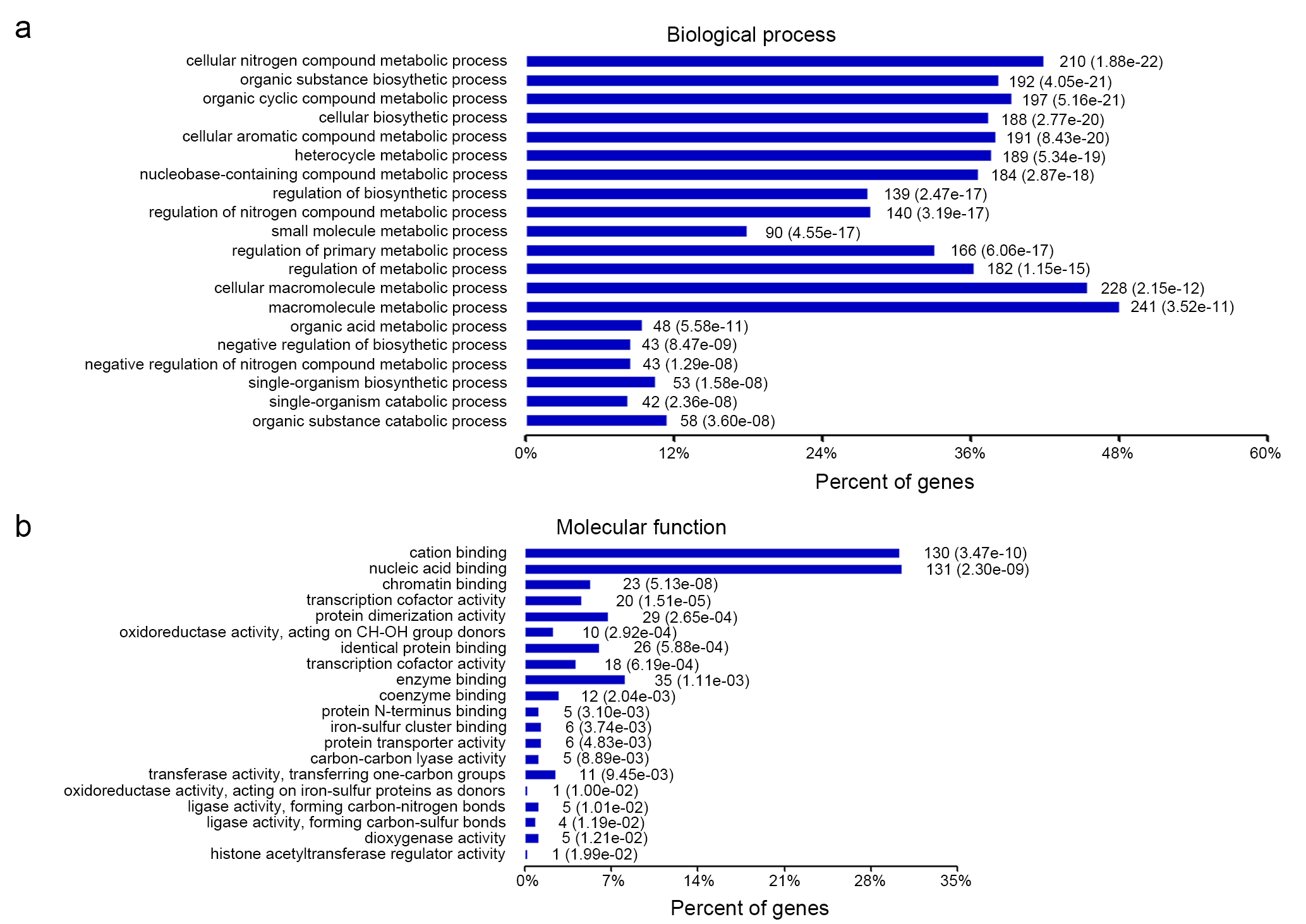

Supplement: Supplementary file 9 — Figure S5. Gene Ontology analysis of genes correlated with B3GALNT2. a Biological process enrichment in the GO analysis of genes correlated with B3GALNT2 in TCGA-LIHC dataset. b Molecular function enrichment in the GO analysis of genes correlated with B3GALNT2 in the TCGA-LIHC dataset. (TIFF 322 kb) [file 13045_2018_595_MOESM9_ESM.tif]

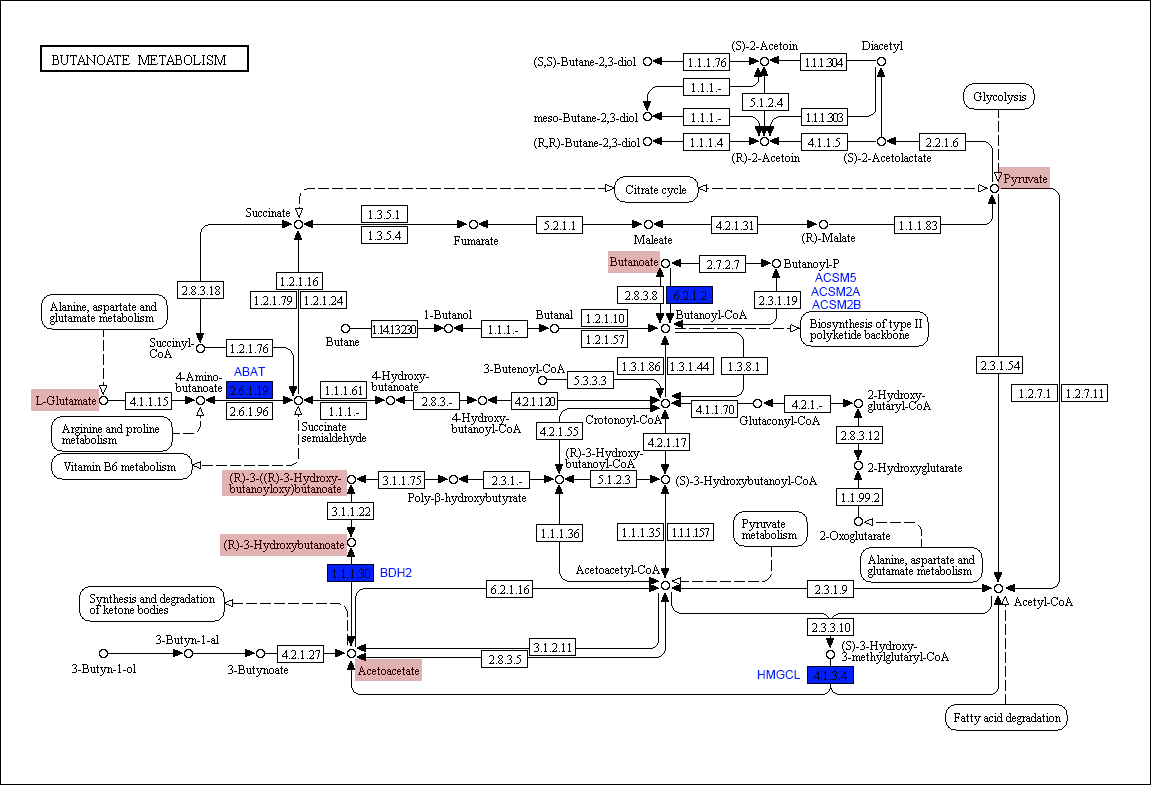

Supplement: Supplementary file 10 — Figure S6. KEGG pathway schematic. KEGG pathway schematic of butanoate metabolism marked with the enzymes and metabolites correlated with B3GALNT2. Blue is for enzymes, red is for metabolites. (TIFF 82 kb) [file 13045_2018_595_MOESM10_ESM.tif]
