# Supplementary material for: Increased B3GALNT2 in hepatocellular carcinoma promotes macrophage recruitment via reducing acetoacetate secretion and elevating MIF activity
Source: J Hematol Oncol. 2018 Apr 4;11:50. doi: 10.1186/s13045-018-0595-3 (PMC5885466; doi:10.1186/s13045-018-0595-3)
Supplement: Supplementary file 2 — Table S1. Relationships between the B3GALNT2expression and the clinicopathological variables of hepatocellular carcinoma patients. (DOCX 20 kb) [file 13045_2018_595_MOESM2_ESM.docx]

**Table S1. Relationships between the B3GALNT2expression and the clinicopathological variables of hepatocellular carcinoma patients**

|  |  | B3GALNT2 Expression | |  |
| --- | --- | --- | --- | --- |
|  |  | high | low |  |
| **Variables** | **No.** | **No. (%)** | **No. (%)** | ***P*-value** |
| **Gender** |  |  |  | 0.107672 |
| Male | 121 | 105(87%) | 16(13%) |  |
| Female | 18 | 13(72%) | 5(28%) |  |
| **Age (year)** | |  |  | 0.077456 |
| >52 | 71 | 64(89%) | 7(11%) |  |
| <=52 | 68 | 54(79%) | 14(21%) |  |
| **vessel invasion** | |  |  | 0.682531 |
| present | 54 | 45(83%) | 9(17%) |  |
| absent | 85 | 73(86%) | 12(14%) |  |
| **Tumor size (cm)** | |  |  | **0.01188** |
| >5 | 81 | 74(91%) | 7(9%) |  |
| <=5 | 58 | 44(76%) | 14(24%) |  |
| **T stage** |  |  |  | **0.00328** |
| t1+t2 | 103 | 82(80%) | 21(20%) |  |
| t3+t4 | 36 | 36(100%) | 0(0%) |  |
| **recurrence** | |  |  | 0.050219 |
| present | 80 | 72(90%) | 8(10%) |  |
| absent | 59 | 46(78%) | 13(22%) |  |
| **liver cirrhosis** | |  |  | 0.294294 |
| present | 85 | 70(82%) | 15(18%) |  |
| absent | 54 | 48(89%) | 6(11%) |  |
| **HBsAg** |  |  |  | 0.98838 |
| positive | 119 | 101(85%) | 18(15%) |  |
| negative | 20 | 17(85%) | 3(15%) |  |
| **HBcAb** |  |  |  | 0.28282 |
| positive | 121 | 103(85%) | 18(15%) |  |
| negative | 18 | 17(94%) | 1(6%) |  |
| **AntiHCV** |  |  |  | 0.54788 |
| positive | 2 | 2(100%) | 0(0%) |  |
| negative | 137 | 116(85%) | 21(15%) |  |
| **TB (umol/L)** | |  |  | 0.208242 |
| ＞20 | 26 | 20(77%) | 6(23%) |  |
| ≤20 | 113 | 98(87%) | 15(13%) |  |
| **ALT (U/L)** | |  |  | 0.675196 |
| ＞50 | 52 | 45(87%) | 7(13%) |  |
| ≤50 | 87 | 73(84%) | 14(16%) |  |
| **AFP (ug/L)** | |  |  | 0.401532 |
| ＞20 | 95 | 79(83%) | 16(17%) |  |
| ≤20 | 44 | 39(89%) | 5(11%) |  |
|  |  |  |  |  |

*p*<0.05 indicates that differences have statistical significance. *Pearson chi-square tests.
