# Supplementary material for: Increased B3GALNT2 in hepatocellular carcinoma promotes macrophage recruitment via reducing acetoacetate secretion and elevating MIF activity
Source: J Hematol Oncol. 2018 Apr 4;11:50. doi: 10.1186/s13045-018-0595-3 (PMC5885466; doi:10.1186/s13045-018-0595-3)
Supplement: Supplementary file 3 — Table S2. Univariate and multivariate Cox regression analysis for overall survival of hepatocellular carcinoma patients. (DOCX 20 kb) [file 13045_2018_595_MOESM3_ESM.docx]

**Table S2. Univariate and multivariate Cox regression analysis for overall survival of hepatocellular carcinoma patients**

| Univariate | | | |  | Multivariate |  |
| --- | --- | --- | --- | --- | --- | --- |
| **Variables** | **HR** | **95%CI** | ***P*-value** | **HR** | **95%CI** | ***P*-value** |
| **Gender** |  |  |  |  |  |  |
| Male vs Female | 1.322 | 0.7280 to 2.402 | 0.3588 |  |  |  |
| **Age (year)** |  |  |  |  |  |  |
| ≥51.5 vs <51.5 | 0.9407 | 0.6106 to 1.449 | 0.7814 |  |  |  |
| **Vessel invasion** |  |  |  |  |  |  |
| Present vs Absent | 1.901 | 0.9890 to 3.653 | **0.054** |  |  |  |
| **Tumor size (cm)** |  |  |  |  |  |  |
| >5 vs ≤5 | 1.931 | 1.037 to 3.598 | **0.0381** | 2.389 | 1.209 to 4.720 | **0.012** |
| **T stage** |  |  |  |  |  |  |
| T3+T4 vs T2+T1 | 2.601 | 1.454 to 4.655 | **0.0013** | 2.438 | 1.482 to 4.010 | **<0.001** |
| **B3GALNT2 expression** |  |  |  |  |  |  |
| High vs Low | 1.938 | 1.126 to 3.337 | **0.017** | 0.468 | 0.252 to 0.872 | **0.017** |
| **Liver cirrhosis** |  |  |  |  |  |  |
| Present vs Absent | 0.9274 | 0.5920 to 1.453 | 0.7419 |  |  |  |
| **HBsAg** |  |  |  |  |  |  |
| Positive vs Negative | 0.5358 | 0.1997 to 1.438 | 0.2154 |  |  |  |
| **HBcAb** |  |  |  |  |  |  |
| Positive vs Negative | 0.882 | 0.3279 to 2.372 | 0.8036 |  |  |  |
| **AntiHCV** |  |  |  |  |  |  |
| Positive vs Negative | 2.104 | 0.1537 to 28.79 | 0.5775 |  |  |  |
| **TB (umol/L)** |  |  |  |  |  |  |
| >20 vs ≤20 | 0.4972 | 0.2330 to 1.061 | 0.0708 |  |  |  |
| **ALT (U/L)** |  |  |  |  |  |  |
| >50 vs ≤50 | 1.372 | 0.7078 to 2.661 | 0.3487 |  |  |  |
| **AFP (ug/L)** |  |  |  |  |  |  |
| >20 vs ≤20 | 1.088 | 0.5653 to 2.095 | 0.8 |  |  |  |
|  |  |  |  |  |  |  |

**CI, Confidence interval; HR, Hazard ratio; *p*< 0.05 was considered to be statistically significant.**
